# Ethnobotany of wild edible plants in Soro District of Hadiya Zone, southern Ethiopia

**DOI:** 10.1186/s13002-023-00588-2

**Published:** 2023-06-02

**Authors:** Mulatu Hankiso, Bikila Warkineh, Zemede Asfaw, Asfaw Debella

**Affiliations:** 1 Department of Plant Biology and Biodiversity Management, College of Natural and Computational Sciences, Addis Ababa University, P.O. Box 1176, Addis Ababa, Ethiopia; 2 Biology Department, College of Natural and Computational Sciences, Hossana College of Education, P.O. Box 94, Hossana, Ethiopia; 3 Ethiopian Public Health Institute, P.O. Box 1242/5654, Addis Ababa, Ethiopia

**Keywords:** Ethnobotany, Nutraceutical plants, Soro District, Wild edible plants

## Abstract

**Background:**

Despite their paramount importance all over the globe in supporting food security, information about wild edible plants is generally patchy. In this study, we investigated the wild edible plants used by the local people in the Soro District of Hadiya Zone, southern Ethiopia. The main purpose of the study was to document and analyze the indigenous and local knowledge of the people on their abundance, diversity, use and management.

**Methods and materials:**

Purposive sampling and systematic random sampling were used to identify informants who can give information about the wild edible plants of the area. Data were collected by interviewing 26 purposively sampled key informants and 128 systematically randomly sampled general informants using semi-structured interviews. Guided observations and 13 focus group discussions (FGDs) consisting of 5–12 participants/discussants at each FGD session were also undertaken. Statistical analyses (mainly descriptive statistics approaches) and common analytical tools of ethnobotany including informant consensus, informant consensus factor, preference ranking, direct matrix ranking, paired comparison and index of fidelity level were applied to the data sets.

**Results:**

A total of 64 wild edible plant species belonging to 52 genera and 39 families were recorded. All of these species are indigenous, 16 are new additions to the database and seven of them, including *Urtica simensis* and *Thymus schimperi*, are endemic to Ethiopia. In about 82.81% of the species, the edible plant part is also used in the Ethiopian traditional herbal medicine. It is striking to see that almost all wild edible plants recorded from the study area are nutraceutical plant species, serving multiple roles as food and therapeutic sources for the local people. We recorded five growth habits of 34.38% trees, 32.81% herbs, 25% shrubs, 6.25% climbers, and 1.56% liana. We found the Flacourtiaceae, Solanaceae, and Moraceae to be families that represented more species (4 each), followed by Acanthaceae, Apocynaceae, Amaranthaceae, and Asteraceae, which accounted for 3 species each. Fruits (53.13%) and leaves (31.25%) were consumed in more proportions than other edible parts (15.63%); mostly the ripe, raw fruit is eaten upon simple processing, followed by leaves eaten after boiling, roasting and cooking.

**Conclusion:**

The frequency and intensity of consumption of these plants varied significantly (*P* < 0.05) with gender differences, key and general informants, and people’s religious backgrounds. We postulate that priority setting for in situ and ex situ conservation of multipurpose wild edible plants in human-inhabited landscapes is essential to warrant sustainable use and conservation of the species as well as the use of new modes of application and valorization.

## Background

In the world, there is an accumulation of ethnobotanical knowledge of wild edible plants (WEPs) that are used for the survival of human life. The use of these essential WEPs has been well documented in different regions of the world, particularly those used more frequently during times of food insecurity [1–3] and in low-income communities. They are a supporting basket of global food (sometimes referred to as the wild supermarket) feeding numerous human populations in situations of various environmental shocks, drought, and famine [4]. Moreover, people mainly living in rural areas depend on different types of wild foods from various growing habitats (from agricultural lands, forest and forest patches, grazing woodlands, permanent and temporary riversides, and the like) based on indigenous culture [4, 5]. WEPs have paramount importance all over the globe for supporting food security [6] to improve the nutritional values, and antioxidants in diets, and this is more so for people in the developing countries [7]. According to the reports of FAO [8], more than one billion people in the world use mostly wild plants as food sources.

Consumption of wild edible plants is one of the feeding habits and features of the community in developing countries including Ethiopia [4, 9, 10]. Different parts of WEPs such as fruits, leaves, roots, tubers, seeds, rhizomes and other parts used for the supply of different food types [4] and used for sources of local tea spices (like leaves with young shoots as observed in *Ageratum conyzoides* and *Dicliptera laxata* in the current study district).

In the plant use habits of the indigenous communities, WEPs also serve as a source of local cash income for the rural communities [11–13]. WEPs could also be used as regular food (sometimes utilized as complementary food) and supplementary foods (i.e., mainly serving wild edibles for children and other indigenous community members. WEPs are important in food/nutrition diversification to complement and balance the modern cereal-dominated nutrient sources [14, 15] including as means for ensuring the food sovereignty of indigenous local communities. However, despite their significance as food and medicine as well as dietary antioxidant sources and as components of community-valued food ingredients, at present traditional knowledge and practices of WEPs are being eroded through acculturation and loss of biodiversity due to human activities. Aboriginal people could be cited as examples to verify the importance of traditional WEPs and the eroded indigenous knowledge, culture and biodiversity loss that led to the challenges in livelihood [16]. Hence more ethnobotanical investigations are very crucial for future societies to maintain and overcome impacting factors of indigenous plants on the ethnobotanical knowledge of the people. Such knowledge can serve to conserve many important WEPs for overcoming a painful period of modern food problems that many countries are facing today.

Despite all these benefits and values, WEPs are not adequately documented in many parts of Ethiopia. So far, information on 413 WEPs under 224 genera in 77 families further shows that these were documented from only about 5% of the 494 Ethiopian weredas/districts [17]. These species are used as seasonal supplementary foods having a potential role to combat food shortage that leads to famine. Another study provided information on 378 WEPs used in Ethiopia [17]. Soro wereda is among those administrative districts not covered in the various reports available to date. A publication by Asfaw and Tadesse [11] had earlier indicated that about five percent of the total WEPs contribute to human food consumption and are utilized during normal periods and in famine situations when the food insecurity challenges escalate [17].

In Ethiopia, the favorable climatic conditions, topographic features, ethnicity, linguistics and religious diversity led to the accumulation of wild plant lore [18]. The study undertaken recently in other parts of Ethiopia showed the indigenous use of plants and the possibility of conserving various multipurpose plants in different agroclimatic settings [19, 20]. Ethnobotanical WEPs are growing in various natural habitats [21]. They are neither cultivated nor domesticated but available in wild habitats and harvested at different seasons to fill the gap of food insecurity [17, 22] and to supplement the regular food at other times [18, 23, 24]. Studies made in parts of southern and western Ethiopia [23–26] have shown that WEPs are important for nutrition, particularly for children and women.

Geographically, Ethiopia is located in the East African phytogeographical region with diverse ethnic groups and biological diversity with enormous traditional practices; many parts of the country are still unexplored or under-explored about ethnobotanical knowledge. Like many parts of Ethiopia, indigenous people in Soro also used wild plants as foods and nutraceuticals in addition to other multiple purposes (*i.e*., different WEP species are used as sources of food and medicine).

Soro District is among the unexplored areas of Ethiopia regarding the ethnobotanical wealth of WEPs. Therefore, this study aims to document the diversity and multipurpose role of WEPs to fill the information gap in the documentation of WEPs and their uses. The mode of transfer and status of the indigenous knowledge of WEPs, the local management system, and threats are also examined.

## Methods and materials

**Fig. 1:**
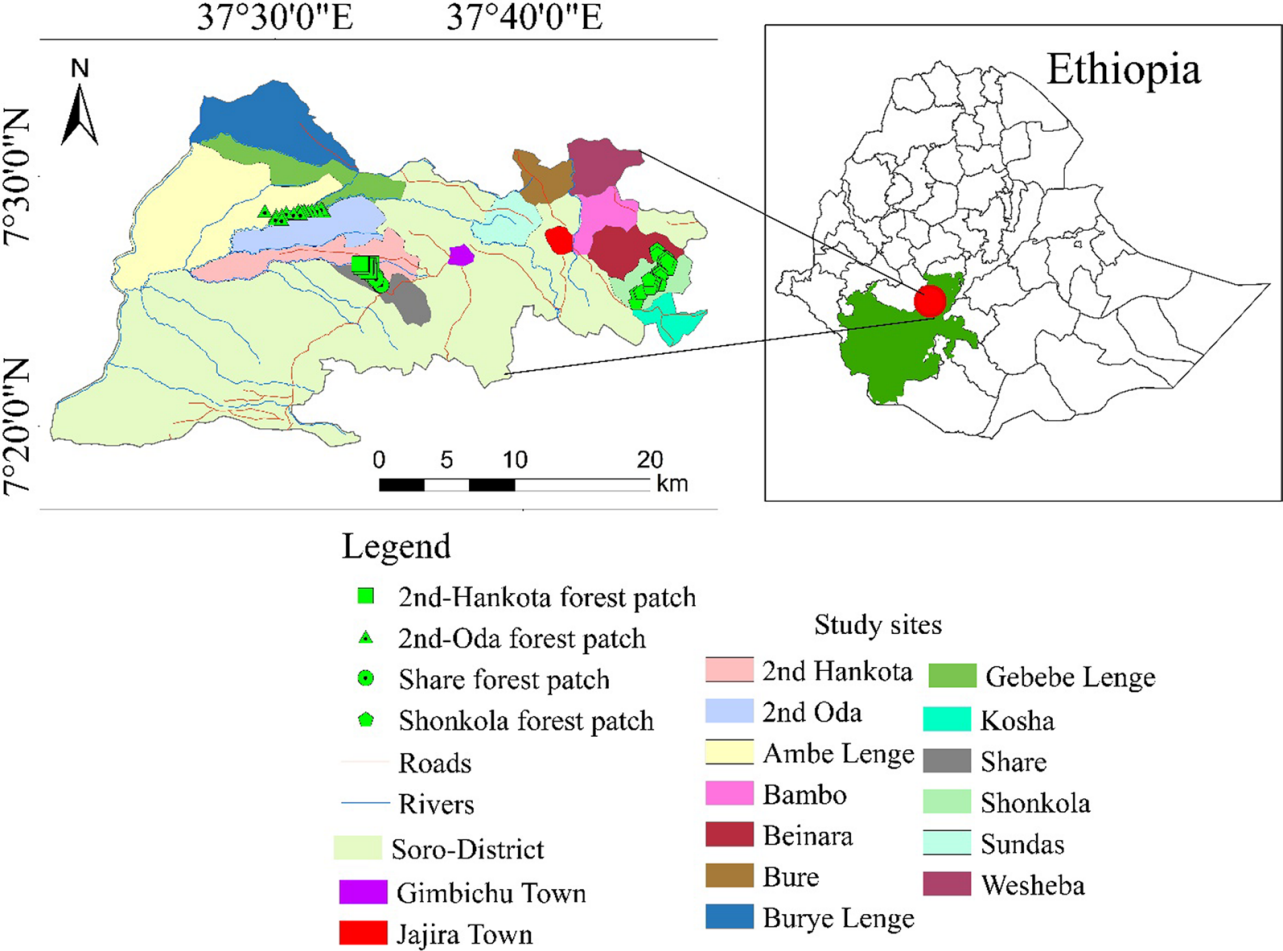
Map of Ethiopia showing the location of Soro District in southern Ethiopia (details of the study sites are given in Table 1)

The field study on WEPs of Soro District was conducted at the time intervals of March 2021–April 2021 and October 2021–November 2021. Major towns in the district include Gimbichu, the center of the district, and Jajura. The district is one of the fifteen districts of the Hadiya Zone, and the people of Soro are Cushitic language (Hadiyissa) speakers of the Hadiya ethnic group. The district is located 32 km away from Hossana town in the southwest (SW) direction, 200 km SW of Hawassa town of Southern Nations, Nationalities and Peoples Region, and 264 km SW from Addis Ababa, the capital of Ethiopia. Geographically, Soro District lies between 37° 20′ 0″ to 37° 47′ 23″ E longitudes and 07° 19′ 4″ to 07° 33′ 48″ N latitudes, with altitude ranges from 799 to 2934 masl. The Kembata Tembaro Zone borders it on the south, the Dawro Zone on the SW, the Omo River on the west, the Duna District on the southeast, the Gomibora District on the north, the Lemo District on the northeast, and the Mountain chains of Gibe River valley on the western lower part nearby Yem Special District [27]. It has features of the Omo-Gibe basin with two tributaries of the Gibe River (Fig. 1).

This study district has 33 rural kebeles with two rural towns of Kosha and Abuna. The total land area covers 36473.337 km^2^ (3647333.7 ha). The population of Soro is 287,589; of these, 143,835 are men and 143,754 are women [28]. The majority (87.42%) live in rural environments, and the rest (12.58%) live in urban centers [28].

The economic activities and livelihoods of the community are agriculture (85%), livestock and crop production, beekeeping and limited commerce [28]. Each household ranges from 0.5 to 2.5 hectares of average agricultural land tenure per farmer household; 10% of the population is employed, 3% merchants and 2% others [28].

The topography is characterized by high mountains of dega/highland (e.g., Mountain Shonkola with its high peak at 2836 masl), surrounding hills, and flat lands. This topographic variation contributes to the diversification of wild food plants. All study information was captured with a map of the study area, a climate diagram, a pie chart, tables and numbers with percentages.

The vegetation of the study area is characterized by the Afroalpine belt (AA), Dry evergreen Afro-Montane Forest and Grassland complex (DAF) and *Combretum-Terminalia* vegetation types that make suitable habitats for various plant compositions and diversity including WEPs. The current vegetation classification of Ethiopia is characterized by the presence of different indicator species [29]. *Lobelia giberroa* and *Erica arborea* are indicator species of the Mountain Shonkola Forest patch of the study area, representing the vegetation type of AA and other representative indicator species of DAF, of which *Carissa spinarum, Euclea divinorum, Syzygium guineense*. subsp. *afromontanum* are WEPs and other wild edibles like *Asparagus africanus, Myrsine africana, Phoenix reclinata, Rubus apetalus, Rubus steudneri, Rumex nervosus, Sideroxylon oxyacanthum, Thymus schimperi, Toddalia asiatica,* among others. The *Combretum-Terminalia* vegetation type includes some representative WEP species such as *Acokanthera schimperi, C. spinarum, Diospyros mespiliformis, Ficus thonningii, Ficus vasta, Oncoba spinosa, Piliostigma thonningii, Maytenus senegalensis, S. guineense* var. *guineense, Warburgia ugandensis*, *Ximenia americana* and *Ziziphus spina-christi.* During different rainy seasons, these vegetation types provide ample supplementary wild edible foods to the community with medicinal and other uses. However, the vegetation of the study area (variously characterized types of remnant forest patches is under the pressure of human activities and mainly agricultural expansion as a consequence of wild edibles, medicinal and extractive use for other purposes, and these resources are declining.

Agroclimatic features of the district are mainly categorized into 39.4% dega (high land), 36.4% woinadeg*a* (middle land) and 24.2% kola (low land) climates. The altitudinal range of the main agroclimatic zones is classified, respectively, into 2300–3500 masl, 1500–2300 masl and 500–1500 masl [30].

Patterns of rainfall distribution and temperature regimes vary within the study area. The rainfall has a bimodal pattern with a short (March–May) and long rainy season that extends from June to August [31], sometimes extending from June to September [27]. According to the District Agricultural Office, the mean annual maximum rainfall is 900–1500 mm which has an opportunity for the growth of common crops. The most extended longest rainy season is summer, traditionally “Kiremt,” which is the time of the main cropping and growing season. The harvesting season is winter (“Bega”). While the short rainy season Mehere (“Belg”); the cropping season of *Zea mays* (Boqqolla-Hadiyissa/Had.), *Solanum tuberosum* (Dinnichcho-Had.), varieties of *Hordeum vulgare* (Gillaloo’i so'o-Had.), *Phaseolus lunatus* (Lob otongora-Had.), *Vigna unguiculata* (Hoffi otongora-Had.) and harvesting in June to replace other cereal crops. For instance, *Vicia faba* (Baaqeela), *Triticum aestivum* (Arasa-Hadiyissa/Had.), *Pisum sativum* (Gite’e-Had.), *H. vulgare* (So'o-Had.), *Eragrostis tef* (Xaafe’e-Had.), *Brassica carinata* (Fiishsho’i shaana/Asussa-Had.).

**Fig. 2:**
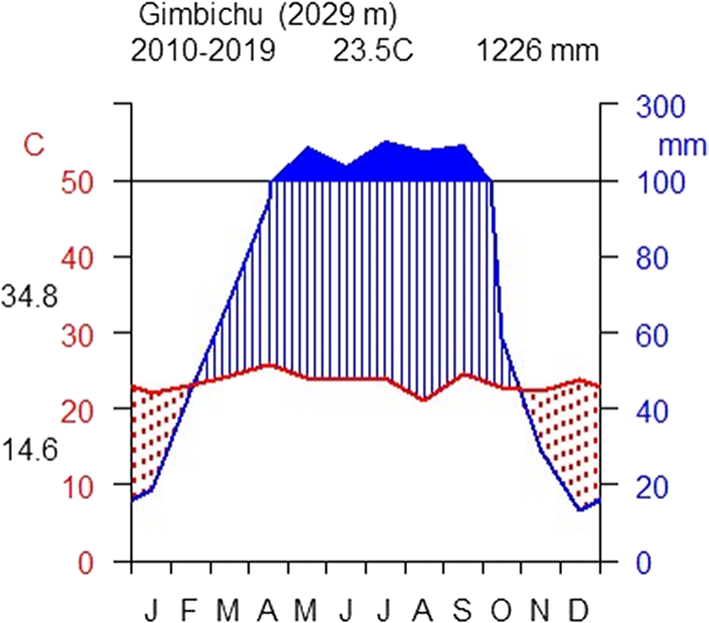
Climate diagram of Soro District, Gimbichu (Data source: NMSA, Ethiopia)

According to climate data (2010–2019) from the center of the National Metrological Services Agency, NMSA, the mean annual rainfall is 1226 mm; the peaks are between March and August and the beginning of September. The yearly mean annual temperature of the district is 23.5 °C (Fig. 2). At the same time, a dry season occurs from November to February. March to April and mid-June, the long rainy season, is also the time of flourishing and ripening wild edible plants. The highest rainfall occurs in July and August, the time of main cropping and growing cereal crops; later, rain decreases in September. According to secondary data, the highest average maximum temperature of the study area in Gimbichu is 34.8 °C in the warmest month. The lowest average minimum temperature is 14.7 °C and is recorded relatively coldest month.

### Site selection and sampling methods

The investigation and data collection of wild edible plants was conducted in three agroclimatic zones in Soro District, southern Ethiopia, at different time intervals during their flowering and fruiting times. Guided observations and reconnaissance surveys were made first before site selection. A focus group discussion was made in Gimbichu town that involved 12 participants (11 males and one female), Soro District, in November 2021.

Different stakeholders were involved from various officers and thirteen potential kebeles were purposively selected from three agroclimatic zones. Each kebele administrator was involved in facilitating the processes of informants selection and FGDs and gave guidance and information on potential vegetation areas where WEPs and their uses are expected to be higher. Four potential sites were identified from four directions for data collection, focusing on wild edible plants with the participation of different informants. The basic data about the study sites (kebeles) including altitudinal ranges, agroclimatic zones and informants’ socio-demographic attributes (gender, ethnicity and language) are given in Table 1. Three of the 13 sites are found in the highland dega agroclimate, seven in woinadega and three in kola. In these sample villages, almost all informants (152; 122 males and 30 females) belong to the Hadiya ethnic group and speak Hadiyissa language; the rest two informants, speak the local language Hadiyissa of the study area and other languages (Afan Oromo and Amharic). Of the interviewed total informants, 141 (111 males and 30 females) are Protestants, 7 Adventists, 4 Apostles and 2 Orthodox. Most of the interviewees (117, 75.97%) were farmers, others housewives (12, 7.79%), non-employed, traders, government employees, unemployed and with no regular income, and farmer and artesian. Most of the informants were married (15198.05%), two widowed and one single. About 81% of the informants had primary school education (grades1–4), (5–6), and 7–8; and 16% secondary school (9–12) and two higher educational level.

**Table 1:** Sampled administrative kebeles with informants interviewed, altitudinal ranges, agroclimatic zone and socio-demographic profile

No	Kebele (Subdistrict)	Altitude (masl)	Agroclimatic zone	Socio-demographic profile			
				Gender		Ethnicity (Had, Oro, Amh)	Language (Hadiyissa, Afan Oromo, Amharic)
				M	F		
1	Kosha	2322–2487	D	8	1	Had	Hadiyissa
2	Shonkola	2321–2826	D	11	2	Had	Hadiyissa
3	Beinera	2186–2453	D	10	1	Had	Hadiyissa
4	Bambo	2061–2111	WD	12	3	Had	Hadiyissa
5	Wosheba	2043–2118	WD	11	4	Had	Hadiyissa
6	Bure	2044–2096	WD	13	3	Had	Hadiyissa
7	Sundusa	2038–2120	WD	10	2	Had	Hadiyissa
8	Share	1755–2062	WD	12	3	Had	Hadiyissa
9	2nd-Hankota	1552–1982	WD	13	4	Had	Hadiyissa
10	2nd-Oda	1705–2097	WD	12	4	Had	Hadiyissa
11	Ambe-lenge	1345–1568	K	6	1	6 Had,1 Oro	Hadiyissa, Afan Oromo
12	Gebebe-lenge	1541–1550	K	2	1	Had, 1 Amh	Hadiyissa, Amharic
13	Burye-lenge	1495–1548	K	4	1	Had	Hadiyissa
Total		–	13	124	30	–	–

*Had* Hadiya/Hadiyissa, *Amh* Amhara/Amharic/Amharigna, *Oro* Oromo/Afan Oromo, *D* Dega, *WD* Woinadega, *K* Kola

### Design of sampling and informant selection

A sample size of the study sites was determined following standard procedure [32] based on the heterogeneity of the three agroclimatic zones having altitudinal variation and agroecology (high land, middle land and low land), potential vegetation areas, forest patches, information on the occurrence of knowledgeable informants, elderly knowledgeable people are known to have rich indigenous knowledge on uses of multipurpose wild edible plants. A total of 154 informants were involved; 128 general informants were taken by systematic random sampling, and purposively selected 26 key informants (two knowledgeable key informants from each kebele) were chosen to get sufficient information about WEPs following the recommended in different literature sources [33, 34].

### Data collection and identification of voucher specimens

Ethnobotanical data of wild edible plants were collected from different elevation sites ranging from 1345 to 2836 masl following the guided field observation, reconnaissance survey and semi-structured interviews of the purposively selected key and systematically random sampled general informants. The key local informants were selected using purposive sampling techniques, which were made at each study site. Market surveys in four markets of the study area (Gimbichu, Jajura, Humaro and Kosha) and focus group discussions using various representatives were made. Voucher specimens were collected from thirteen kebeles of selected sites with the help of local field guides and information from the FGDs. Collection sites include home gardens, agricultural lands, roadsides, forests, grasslands, and river sides/margins. Notes on growth form, living habitat and other particular features of each plant were recorded. Identification of common and easily known voucher specimens was made in the field. Specimens of all plants recorded (identified and unidentified) were brought to the National Herbarium [ETH], Addis Ababa University, and identified, confirmed, and standard labels were prepared following the usual herbarium techniques [35]. For example, the scientific names of the species collected were determined using the relevant volumes of the Flora of Ethiopia and Eritrea [36, 37]. The determination was further refined with visual comparison using authenticated herbarium specimens, and finally, the accuracy was checked by a senior plant taxonomist. The plant specimens with their labels were finally deposited at the National Herbarium (ETH) in Addis Ababa, Ethiopia.

### Focus group discussions (FGDs)

During actual data collection, 5–12 participants were involved in focus group discussions representing various groups of people. One FGD was conducted in each kebele using semi-structured questions where knowledgeable cattle-keeping young children, kebele managers, key informants, community elders, religious and community leaders, forest patch dwellers, apiculturists and woodworkers, potter’s men, and women were participating. They responded to questions on the diversity of wild edible plants, most preferable WEPs, common and rare edibles, threats to wild edible plant species, and ways of conservation and management. Moreover, participants provided information about using wild edibles and helped collect specimens. Each discussion was guided by the kebele administrator, guide and environmental protection expert, and forest and climate change officer, who also served as language translators for other team members’ discussions. Verbatim information from the meeting was chaired and recorded by the investigator (first author). Local names of wild edible plants, parts used, maturity level used for consumption, seasons/months of ripening, consumption time (during a shortage of regular food such as during drought and famine or normal periods), how and who prepares more using different preparation methods for consumption, causes of health problems and feelings if occurred when the parts are consumed; antidote and other uses were discussed.

A total of 113 FGD participants in 13 kebeles, 12 males and 25 females aged 18–35; 31 males and 20 females aged 36–59, and 22 males and three females aged > 60 years were involved. Different numbers of participants in each FGD were involved.

### Methods of data analysis

Gathered data were analyzed by qualitative and quantitative approaches, and descriptive statistics [33]. Microsoft Excel spreadsheet software version 2016, SPSS version 25, and one-way ANOVA and R program using R.4.2.2 software were employed for the analysis of certain ethnobotanical data. Informant consensus and ICF, preference ranking, direct matrix ranking, paired comparison and index of fidelity level were conducted for data analysis through crosschecking and verification of the information.

### ICF (Informant consensus factor)

Informant consensus describes the agreement between respondents when choosing the most cited specific wild edible plant species (Table 2). It was used to evaluate and prioritize the reliability of the edible plants. Also, the informant consensus factor values were calculated by applying the number of citations of individual species minus the number of selected species [38]. It was calculated to check in-between 0 and 1, based on the number of each selected wild edible plant species use citation (Nur), which accounted for 40, and the number of selected species used (Nt) was 12. Thus, the ICF number is 0.72, and the product is greater than zero but close to one, which informed that various WEP species are used for multipurposes in the indigenous community.

**Table 2:** Distribution of WEPs in different agroclimatic habitats

Habitat of collection	Agroclimatic zones	No. of species collected	%
Family home garden, HG	Dega, woinadega and kola	20	31.25
Live fence and/or dry fence (Lf and/or Df)	Dega, woinadega and kola	2	3.13
Roadsides (RS)	Dega and woinadega	3	4.69
Forest patches (FPs)	Dega, woinadega and kola	25	39.06
Agricultural/farm lands (AL)	Dega, woinadega and kola	3	4.69
Riverine/River valley/areas, Ria	Dega, woinadega and kola	6	9.37
Grass/bush land (GL/BL)	Dega, woinadega and kola	5	7.81
Total	–	64	100

### Preference ranking

Simple preference ranking was made by arranging a rank of the most preferred as well as popular ethnobotanical wild edible plants following common sources [34]. Key informants were used to assess the degree of preference for edible fruits and leaves highly cited by informants. Based on the total score of each species, the rank was determined by the informants’ preference.

### Direct matrix ranking

The direct matrix ranking (DMR) method was conducted for multipurpose use values of wild edible plants commonly reported by key informants [34, 39]. DMR is one of the multifaceted types of preference ranking techniques. Based on the relative benefits obtained from each chosen ten plant species, ten key informants were asked to assign values by giving order to each attribute among different uses such as medicinal, wild food, fodder, construction material, timber production, farming tools, utensils, firewood, fuels, shade, and live fence. Each chosen informant was asked to assign use values (5 = best, 4 very good, 3 good, 2 = less used, 1 = least used and 0 = not used). The average values of a score of each species were summed up and ranked. By adding the score values, it was possible to assess the relative importance and to check the major impacts due to the higher exploitation of each plant species than other species in the study site. Such data could be used for setting conservation priority.

### Paired comparison

The paired comparison method was used to determine the relative importance of some WEPs to evaluate the degree of use and community preference as edibles. Eight WEPs were paired to compare individual respondents to each other, and decisions were made by individual respondents on the relative importance of one edible plant from a pair  [33]. A couple was chosen by some of the four key and four general informants (Table 6). The total number of possible pairs was obtained by the formula: $n = \frac{{n\left( {n - 1} \right)}}{2}$, where n is the number of important WEPs being compared. For this exercise, equal numbers of informants were randomly involved (4 key and 4 general informants).

### Index of fidelity level (FL = Ip/Iu × 100)

Index of fidelity level (FL) is a commonly used method to quantify, compare and determine the relative importance of a plant species for a given function [34], using the following formula: where Ip is the number of informants who independently cited the importance of a species for a particular purpose and Iu is the total number of informants who reported the plant for any given use. The knowledge comparison on WEPs based on age, gender, educational status, key and general informants, and an agroclimatic zone among various socio-demographic groups in the study area was also computed.

## Results

### Diversity of wild edible plants (WEPs) in Soro District

In this research, a total of 64 species of WEPs that belong to 52 genera and 39 families were documented. Further analysis showed that the family Flacourtiaceae had 4 (6.25%) species in 3 (5.77%) genera, Solanaceae 4 (6.25%) species in 2 (3.85%) genera, and Moraceae 4 (6.25%) in 1 (1.92%) genus. Other 36 families contributed 52 (81.25%) species distributed in 46 (88.46%) genera. These WEPs were collected from various habitats of forest patches, riverine areas, grasslands, agricultural lands, roadsides and homegarden yards with or without cultivated crops. Some wild edibles are cultivated by households in association with other naturally growing wild useful plants.

The records also included collected edibles, most of them used for herbal medicines with nutraceutical values in addition to supplementary as well as regular wild edible food sources during drought and famine which support human food security. For example, the roasted or cooked leaves and young shoots of *Amaranthus dubius* and *Bidens pachyloma* were mostly used during famine and consumed like some cultivated species of leafy vegetables such as *Brassica oleracea* var. *oleracea* and *B. carinata*, and the fruits of *Ficus sycomorus, Ficus sur* and *O. spinosa* are eaten by removing the exocarp, whereas the tuber of *Dioscorea schimperiana* is eaten as corm of *Ensete ventricosum* and tuber of *S. tuberosum* by cooking and peeling the thin exocarp.

FGD participants explained their observations that the diverse wild edibles are eaten more as snacks/refreshments and supplementary as well as regular wild food sources during food insecurity. A good number of the species are also said to have traditional medicinal and other uses. The WEPs provide edible fruits, leaves with terminal and lateral shoots/buds, tubers, and other parts used as chewing gum and spices of tea by the society. They are consumed by picking raw ripe fruits and mature leaves. Common examples in the study area are *F. sur, F. sycomorus, S. guineense* subsp. *afromontanum, S. guineense* var. *guineense, W. ugandensis, Landolphia buchananii, C. spinarum, X. americana, Flacourtia indica, T. asiatica* and *P. thonningii.* Some WEPs are eaten as regular wild food through rarely and other dominants are eaten as supplementary foods, in the study area, households and individuals during food shortages (e.g., *F. sycomorus*, *F. sur,* *A. dubius, D. schimperiana, B. pachyloma and O. spinosa)*.

Specimens of these and other non-crop ethnobotanical edible plants were reported and collected from wild areas of dega, woinadega and kola agroclimatic zones within the altitudinal rages of 1345–2836 masl. Wise use of the above-explained results of edible leafy vegetables, tubers and fruits could ensure the sustainable availability to ensure the presence of food security as well as food sovereignty in the local community of the study area. However, today a large number of proportions of the population do not consume wild plants due to high dependency on staple food crops and they used wild edibles as accessory food sources.

### Growth habits of wild edible plants

**Fig. 3:**
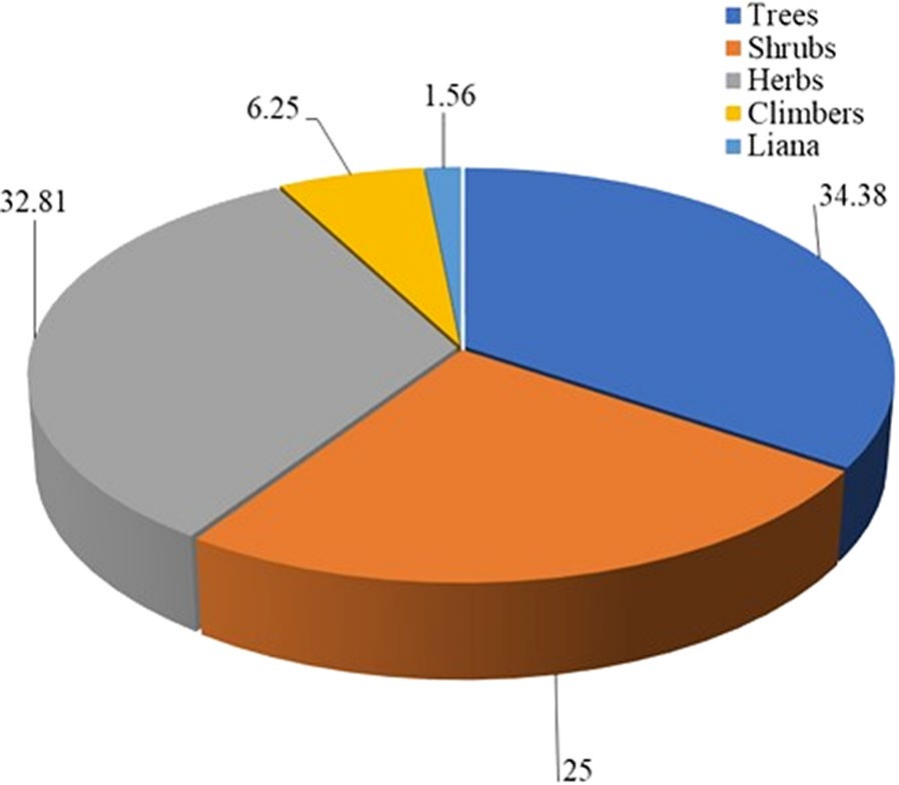
Growth habit (life form) of wild edible plants in Soro District

Of the total WEPs, trees took the highest growth form and proportion 22 (34.38%), liana took the least life form 1 (1.56%), whereas herbs (21) were the next highest life form followed by shrubs (16) and 4 climbers (Fig. 3). Trees were also categorized into 16 families and 18 genera, herbs 13 families and 17 genera, shrubs 11 families and 15 genera, and climbers 4 families and 4 genera. In contrast, liana had the least one family and one genus (Table 10).

### Frequency of wild edible plant parts and their proportion

Out of the total reported and eaten parts of wild edible food resources, fruits contributed the most significant palatable amount and percentage, accounting for 34 (53.13%) species, leaves follow in the second place as edible part, and the proportion accounted for 20 (31.25%) species (Table 2). Species known for having edible fruit and gum accounted for 5 (7.81%), leaf and fruit 2 (3.13%), tuber 2 (3.13%), and flower–inflorescence nectar accounted for 1 (1.56%). Hence, fruits and leaves are the most dominantly consumed and widely used edible wild plant parts in the study area, respectively. Of the wild edible plants, five species (*F. sur, F. sycomorus*, *F. thonningii, F. vasta* (Moraceae) and *L. buchananii* (Apocynaceae) also produced milky latex used as, chewing gum, as a plastering material and sipping sap (*e.g., L. buchananii)*. *Landolphia buchananii* is used for making traditional play balls with parasitic mosses using milky latex that is produced from stem bark when cut or injured the bark.

Of the total reported WEPs, 34 are fruits; of them, 16 (47.06%) trees, 12 (35.29%) shrubs, 3 (8.82%) herbs, and 3 (8.82%) climbers. Of the whole leaf edibles (20), 2 (10%) trees, 3 (15%) shrubs, and 15 (75%) herbs contributed more proportion than trees and shrubs edibles. From 5 fruits and gum consumption plants, 4 (80%) are trees, and only 1 (20%) contributed liana; of the two leaf and fruit edibles, both species are herbs (100%); from 2 tubers category herb and climber contributed 1 (50%) each.

### Mode of consumption

The people in the Soro District consume plants in raw ripe form without processing or upon cooking or roasting. The majority (68.75%) of the species are harvested and used in raw mature form by cleaning the dirty, washing the edible parts with clean water, and removing thick or thin non-edible epicarp and some hard stone endocarp seeds. Some 31.25% were eaten upon processing by chopping with a knife, and some were roasted or cooked using local clay pots and metallic cookers. In a few ripe raw wild edible plants, stems of some plant species are injured or cut. Sweet-tasting latex is released out and sipped by herd of cattle-keeping children and also used for chewing gum by painting or smearing the milky latex on the hand and allowed to dry (e.g., stem latex of *L. buchananii*, *F. sur* and *F. sycomorus*).

### Marketability of wild edible plants in Soro District

People interviewed in the local markets informed that for two decades few species were sold to generate cash income, as is the case with *S. guineense* var. *guineense*, *S. guineense* subsp. *afromontanum* and seeds of *Amaranthus caudatus* was mainly sold as a food source in dega and woinadega, and rarely *C. spinarum in* kola agroecological settings. The community, in their habitats, consumed various wild food species in ripe and raw form. FGD informants also reported few WEPs were sold in the local markets to generate local income; they used the above two fruit edibles, seeds and rarely fruit of *C. spinarum* in kola agroecology.

### Informant consensus on the most repeatedly and frequently reported WEPs

Certain wild edible plants were commonly used in the study area as the source of supplementary and regular wild food during food insecurity/famine than other wild food plants. As a result of this, the ripe raw fruit and leaf with shoot edible plants frequently reported as a source of stable food were *F. sycomorus*, reported by 147 informants and eaten fruits, *A. dubius* reported by 140 informants, and eaten leaf with shoots, *D. schimperiana* was reported as, regular wild edible by 138 informants and eaten tuber, and *O. spinosa* was reported 136 as regular food and fruit eaten, *S. guineense* subsp. *afromontanum* by 125 informants and eaten fruits as supplements, and *B. pachyloma* reported by 118 eaten leaves as regular with young shoots. The remained others were preferable supplementary wild edibles and all of them are potential plants for food security as well as food sovereignty in the study area for future food scarcity due to drought (Table 3).

**Table 3:** Informant consensus of most commonly eaten fruits and leaves with shoots eaten plants

Scientific name	Part(s) eaten	No. of Informants	%	Diet
*Ficus sycomorus*	Ripe raw fruits	147	95.4	Reg
*Amaranthus dubius*	Roasted/cooked leaves	140	90.9	Reg
*Dioscorea schimperiana*	Mature tuber cooked	138	89.6	Reg
*Oncoba spinosa*	Ripe raw fruits	136	88.3	Reg
*Warburgia ugandensis*	Ripe raw fruits	134	87.0	Sup
*Landolphia buchananii*	Ripe raw fruits	132	85.7	"
*Carissa spinarum*	Ripe raw fruits	129	83.8	"
*Syzygium guineense* subsp.* afromontanum*	Ripe raw fruits	125	81.2	"
*Ximenia americana*	Ripe raw fruits	123	79.9	"
*Piliostigma thonningii*	Raw and cooked leaves	119	77.3	"
*Bidens pachyloma*	Cooked leaves and shoots	118	76.6	Reg
*Amaranthus caudatus*	Cooked leaves and seeds	115	74.7	"

*WEPs* Wild edible plants, *Sup* Supplementary food [**"**], *Reg* regular wild food by a large proportion of the local community

The number of ICFs (0.72) which resulted in greater than 0, approximately 1, showed that different WEPs are used for various purposes for the local people who lived in the community in addition to food.

### Preferences for some WEPs

The key informants ranked 13 edible fruits based on the degree of preferences among the highly cited wild edible plants in the Soro District. The wild edible fruit most preferred by the community scored “13” and the lowest score “1,” others being in between (Table 4).

**Table 4:** Simple preference ranking (SPR) values of the most commonly used top 13 wild edible fruits

Botanical name	Respondents														
	R_1_	R_2_	R_3_	R_4_	R_5_	R_6_	R_7_	R_8_	R_9_	R_10_	R11	R12	R13	Total	Rank
*Ficus sycomorus*	13	6	7	8	7	6	9	8	7	4	10	5	6	96	1
*Landolphia buchananii*	10	13	8	7	6	5	6	7	5	7	8	5	7	94	2
*Syzygium guineense* var.* guineense*	5	10	7	6	9	8	13	11	5	8	9	1	1	93	3
*Warburgia ugandensis*	8	7	8	5	7	6	9	5	7	12	5	4	6	89	4
*Oncoba spinosa*	5	8	5	11	6	6	5	10	5	6	4	3	7	81	5
*Ficus sur*	7	6	8	5	7	5	9	5	6	5	1	4	10	78	6
*Carissa spinarum*	6	7	6	3	6	5	4	5	6	6	12	8	2	76	7
*Passiflora edulis*	9	4	9	1	6	5	8	5	7	6	5	7	2	74	8
*Physalis peruviana*	6	1	5	4	11	6	4	5	6	1	7	8	6	70	9
*Flacourtia indica*	5	6	4	6	1	7	5	4	1	5	8	7	5	64	10
*Toddalia asiatica*	4	6	1	7	5	6	1	7	2	5	7	4	6	61	11
*Rubus steudneri*	1	5	5	2	4	5	2	9	6	5	7	5	3	59	12
*Rubus apetalus*	5	3	2	5	4	1	5	1	3	5	7	5	4	50	13

The top thirteen (13) are the most preferable wild edibles based on the criteria of availability in common in the locality, pulp content,  organoleptic properties of tastes, smell, flavor, and other features, size of non-edible seeds and thickness of exocarp and one (1) is the least with relative to others. Among the compared WEPs, fruit edibles *F. sycomorus* was scored the highest and scored first (SPR = 96) based on fleshy pulp with very small seeds and better sensation of the flavor and used as regular wild food. *Landolphia buchananii* scored second (SPR = 94) and was used as supplementary wild food with better flavor and pleasant taste; *S. guineense* var. *guineense* was the third score (SPR = 93) and supplementary with suitable better taste, *W. ugandensis* scored fourth (SPR = 89) used as supplementary with light white flesh pulp and sweet taste and *O. spinosa* was scored fifth (SPR = 81); it has dense dark brown pulp with small sized berry seeds, with better flavor, used as regular wild food and others were scored and ranked accordingly.

In another comparison among preferable leaf edibles using ten key informants, *Solanum nigrum* (SPR = 75), *A. dubius *(SPR = 73), and *B. pachyloma* (SPR = 69) were the scored highest, second and third preference ranking scores (SPR); others supplementary wild edibles of *A. caudatus, P. thonningii, Solanum* sp*, Urtica urens*, *Commelina benghalensis*, *Rumex abyssinicus* and *R. nervosus* were ranked, respectively, with the SPR values of 68, 56, 53, 50, 48 and 37 and used as supplementary food (Tables 5 and 6).

**Table 5:** Direct matrix ranking score of 10 key informants of nutraceutical plant species with various other uses in Soro District based on use value criteria (5 = for the best, 4 = for very good, 3 = for good, 2 = for less used, 1 = for the least used and 0 = for no use category/value)

Nutraceutical plant species	Use categories/values									Ff	Total	Rank
	Fw	WE	Con	TP	M	Ch	Fo	Ft&Ut	Sha			
*Cordia africana*	5	3	5	5	4	3	1	4	3	4	37	1st
*Syzygium guineense* spp*.*	5	4	5	0	3	4	2	3	5	2	33	2nd
*Warburgia ugandensis*	4	4	5	1	3	4	0	4	5	2	32	3rd
*Mimusops kummel*	5	4	5	0	3	4	1	3	5	1	31	_4_th
*Balanites aegyptiaca*	5	5	4	0	3	3	3	2	3	1	29	_5_th
*Phoenix reclinata*	4	0	5	0	4	4	5	1	1	1	25	6th
*Ximenia americana*	3	2	3	0	5	3	3	2	1	1	23	_7_th
*Trichocladus ellipticus*	5	1	4	0	2	2	4	2	0	1	21	8th
*Moringa stenopetala*	2	0	0	0	5	4	2	1	1	1	16	9th
*Bidens pachyloma*	2	0	0	0	2	4	4	0	0	0	12	10th
Total	40	23	36	6	34	35	25	22	24	14	266	
Rank	1st	7th	2nd	10th	4th	3rd	5th	8th	6th	9th		

Where *Fw* Firewood, *Ch* Charcoal, *Con* Construction, *TP* Timber production, *M* Medicine, *WE* Wild edible, *Fo* Fodder, *Ft & Ut* Farming tool and house utensils, *Sha* Shade, *Ff* Fire formation. The output of the DMR score of ten key informants for ten use diversities showed that some multipurpose wild edible plant species were more highly exploited for firewood, construction, and local charcoal than the other uses. As a result, 1st (*Cordia africana),* 2nd (*Syzygium guineense* var. *guineense* and *Syzygium guineense* subsp*. afromontanum*) *and* 3rd (*Warburgia ugandensis)* ranked plant species become locally extincting and endangering due to unwise use for different functions

**Table 6:** Result of paired comparison of eight wild edible plant species used by the people in the study district

WEP species	Mixed 4 key and 4 general informants R1-R8								Frequency	Rank
	As	Vv	Da	Ea	Ed	Rv	Rn	Hd		
*Acokanthera schimperi* (*As*)	–	Vv	As	As	As	As	As	Hd	5×	3rd
*Vangueria volkensii (Vv)*		–	Vv	Ea	Vv	Ea	Vv	Hd	4×	4th
*Dovyalis abyssinica (Da)*			–	Ea	Ed	Da	Da	Hd	2×	6th
*Erucastrum arabicum (Ea)*				–	Ea	Ea	Ea	Hd	6×	2nd
*Euclea divinorum (Ed)*					–	Ed	Ed	Hd	3×	5th
*Rhus vulgaris (Rv)*						–	Rv	Hd	1×	7th
*Rytigynia neglecta (Rn)*							–	Hd	0×	8th
*Habenaria decumbens (Hd)*								–	7×	1st

The result of the pairwise ranking comparison (PWRC) edibility ranks 1–4 were the most indication of edible species concerning other compared species. The ranks 7 and 8 were relatively preferable important edibles even if they scored fewer ranks

The top ten potential WEPs were quantified using the index of fidelity level (Table 7). The ripening and availability of these species varied and were mainly used to fill gaps in food shortages. *F. sycomorus*, *A. dubius*, and *D. schimperiana* had higher scores and were used as more regular wild food sources including *O. spinosa*, whereas *L. buchananii*, *C. spinarum*, *X. americana* and *P. thonningii* were reported for use as supplementary wild food sources. Both *W. ugandensis* and *S. guineense* subsp*. afromontanum* were noted for being used as additional and regular wild edibles, particularly during famine and the food insecurity gaps. These species required sustainable use and conservation priority in the community of the study area.

**Table 7:** The relative value of fidelity level for potential wild edible plants in the study area

Scientific name	Wild edibles used as supplements or regular wild food	IP	IU	FL	FL%	Rank
*Ficus sycomorus*	Used during the famine as regular wild food	45	45	1	100	1
*Amaranthus dubius*	Used during the famine as regular wild food	29	30	0.90	90	2
*Dioscorea schimperiana*	Used as regular wild food	25	28	0.89	89	3
*Landolphia buchananii*	Used as supplementary wild food	19	22	0.86	86	4
*Oncoba spinosa*	Used as regular wild food	23	27	0.85	85	5
*Warburgia ugandensis*	Used to supplementary and regular wild food	21	25	0.84	84	6
*Syzygium guineense* subsp*. afromontanum*	Used supplementary, more during the famine as regular wild food	14	18	0.78	78	7
*Carissa spinarum*	Used as supplementary wild food	15	20	0.75	75	8
*Ximenia americana*	Used as supplementary wild food	10	14	0.71	71	9
*Piliostigma thonningii*	Used as supplementary wild food	9	13	0.69	69	10

### Indigenous knowledge distribution in different socio-demographic members

More WEPs were reported by male informants on average (12.52 ± 6.07); frequently reported/cited 1553 (84.68%) of all respondents reported WEPs (1834) than females (9.37 ± 3.62); cited 281 (15.32%); and the statistical comparison is also significant (*P* < 0.05). This is usually because males are movable from one area to another, and they had opportunities to gain and share as well as quote more indigenous knowledge about WEPs use than females (owners of households). Although comparing gender informants for indigenous knowledge practices based on WEPs’ preparation for feeding, women are better knowledgeable and familiar with food preparation and cooking, caring for their families and children in their house than men. Key informants frequently reported 474 (25.84%) species, and general informants 1360 (74.15%) frequent species, and significantly varied (*P* < 0.05) at the mean average number (18.23 ± 6.43).

On the other hand, there were insignificant differences (*P* > 0.05) occurred among educational status, agroclimatic zones, and age categories even if a more frequent number of WEPs were reported and accounted by literates 1044 (56.92%) than illiterates 790 (43.07%); woinadega 1318 (71.86%) than dega 365 (19.90%) and kola 151 (8.23%). As well as more indigenous knowledge of WEPs was also frequently reported by old ages (> or = 60 years); 735 (40.07%) and an ages in-between adult ages (36–59 years); 635 (34.2%) than in-between young ages (18–35 years); 464 (25.30%). It might be due to less knowledge transfer among the 26 key and 128 general interviewed informants.

Based on the distance of informants relative to the main town, frequently more WEPs 1376 (75.03%) were reported from far rural areas (> or = 5 km) than nearby 458 (24.97%) of (< 5 km), indicating that people were more relation with the plants and more vegetation availability in the far rural community than urban. The frequently reported result indicated that more varieties of WEPs can be available in rural communities due to potential vegetation sites along forest patches away from an urban community. Statistical it also varied insignificantly (*P* > 0.05) with distance from the town.

Male informants reported more information on WEPs with ethnobotanical knowledge than females and varied numbers of wild edible plants; key informants have rich knowledge information on wild food plants than general informants when they computed their knowledge (Table 8). Frequently more species were reported from woinadega than dega and kola agroclimatic zones, as well as the distance from the town or population center was more in localities closer to natural forests and woodlands compared to urban areas.

**Table 8:** Statistical test of significance using one-way ANOVA the average number of reported WEPs among various variables in the study area, Soro District

Participants	Informants group	*N*	Average ± SD	*F* value	*P* value
Gender	Males	124	12.52 ± 6.07	7.46	0.007
	Females	30	9.37 ± 3.62		
Educational status	Illiterate	65	12.02 ± 5.91	0.04	0.847
	Literate	89	11.83 ± 5.76		
Age category	18–35(Young)	39	11.90 ± 5.07	1.00	0.369
	36–59(Adult)	57	11.14 ± 6.23		
	> 60(Old/Elderly)	58	12.67 ± 5.82		
Proximity to the main town Informants’ category	< 5 km (Urban/Near)	36	12.72 ± 5.92	0.92	0.338
	> or = 5 km (Rural/Far)	118	11.66 ± 5.77		
	Key informants	26	18.23 ± 6.43	48.71	0.000
	General informants	128	10.63 ± 4.75		
Agroclimatic zone	Dega dwellers	33	11.06 ± 4.44	1.55	0.215
	Woinadega dwellers	106	12.43 ± 6.03		
	Kola dwellers	15	10.07 ± 6.56		
Religion	Protestant	141	11.40 ± 5.40	6.01	0.001
	Orthodox	2	11.00 ± 1.41		
	Adventist	7	17.29 ± 8.30		
	Apostle	4	20.75 ± 5.56		

A significant difference (*P* < 0.05); insignificant (*P* > 0.05), *df* = *N*-1;153, *N* = number of respondents = 154

Insignificant differences (*P* > 0.05) occurred in the number of wild edible plants reported by illiterate and literate; young, adult, and elderly; near and distant. Numerous WEPs were frequently reported from dominant informants living in areas from woinadega agroclimatic zone. Key informants reported more ethnobotanical knowledge on WEPs relative to the general and age-old, middle and younger; rural dwellers than urban.

### Nutraceutical wild edible plants

Of the 64 WEPs, 53 were reported for traditional medicine to treat one or more than one human and livestock ailments (Table 9). Leaves were reported the highest numbers (54.02%) by informants’ citations, followed by roots (18.97%), and other proportions of small use citations accounted for 27.01%, which include fruit, actively growing buds, stem bark, aboveground and belowground part, whole part, latex, and resin. Due to the widely used conventional medicinal plants by indigenous people, fresh leaves with buds were dominantly used, followed by fresh roots and fruits. Of the total WEPs, 53 nutraceutical plant species were used to treat 70 human ailments, 18 livestock ailments, and 5 for human and livestock ailments. One or a combination of two or more wild edible plants treated those ailments.

**Table 9:** Some nutraceutical wild edible plants used for the treatment of human livestock ailments

Edible plants with medicinal use reports	Disease	Human/Livestock	Part used in TM in the study area	Part used as food in the study area	Reference for use in TM
*Ageratum conyzoides*	Diarrhea	Human	Leaf	Leaf	[53]
*Amaranthus caudatus*	Michi	Human	Seed	Seed	[12, 53]
	Cancer	Human	Leaf	Leaf	[12, 53]
*Balanites aegyptiaca*	Epistaxis (Nasal bleeding), Headache, Stomach ache	Human	Resin, Fruit	Fruit	[54]
*Bidens pilosa*	Internal cancer, Michi	Human	Leaf	Leaf	[54]
	Constipation	Human	Aboveground	Aboveground	[54]
	Malaria	Human	Aboveground	Aboveground	[54, 55]
*Carissa spinarum*	Amoebiasis, Swelling	Human	Stem bark, Root, Fruit	Fruit	[53]
	Swelling	Livestock	Root, Fruit	Fruit	–
*Moringa stenopetala* (Naturalizing in the wild habitat having been brought from another area*)*	Malaria	Human	Leaf, Root	Leaf	[56, 57]
	Hypertension	Human	Leaf	Leaf	
	Diabetes mellitus (DM)	Human	Leaf	Leaf	[53]
	Mumps	Human	Leaf	Leaf	
	Abdominal pain, Stomach ache	Human	Leaf	Leaf	[53]
*Solanum nigrum*	Heart disease	Human	Aboveground	Aboveground	
	Breast cancer, Skin cancer	Human	Leaf	Aboveground	–
	Internal cancer	Human	Leaf	Leaf	
	Ascariasis	Human	Leaf	Leaf	[53]
	Abdominal pain Stomach ache	Human	Leaf	Leaf	[53, 56]
	Conjunctivitis/Eye disease	Human	Leaf	Leaf	–
	Pityriasis	Human	Leaf	Leaf	–
*Ximenia americana*	Common cold and head ache	Human	Leaf, Stem bark	Fruit	[4, 57]
	Lumpy skin disease (LSD)	Livestock	Leaf, Stem bark	Fruit	[56, 58]
	Tooth disease	Human	Fruit	Fruit	[55–57]
	Abdominal pain Stomachache	Human	Fruit	Fruit	[55, 56]
	Pyelonephritis	Human	Stem bark	Fruit	–
	Diarrhea	Human	Leaf, Root bark	Fruit	–
	Spleen enlargement	Human	Root bark	Fruit	–
	Malaria	Human	Root bark	Fruit	[57]
	*Aspiration pneumonia*	Livestock	Root bark	Fruit	–
	Pyelonephritis	Human	Leaf	Fruit	–

Some species (1) *A. africanus, (*2) *C. spinarum,* (3) *Cordia africana*, (4) *X. americana*, (5) *S. nigrum,* (6) *Physalis peruviana,* (7) *O. spinosa,* (8) *T. asiatica,* (9) *L. buchananii,* (10) *Moringa stenopetala,* (11) *Erucastrum arabicum,* (12) *F. sur*, (13) *F. sycomorus,* (14) *S. guineense* var. *guineense* (15) *S. guineense* subsp. *afromontanum*, (16) *D. schimperiana*, (17) *Balanites aegyptiaca,* (18) *Dovyalis abyssinica*, (19) *P. reclinata*, (20) *P. thonningii*, (21) *Trichocladus ellipticus* and (22) *W. ugandensis* were the most commonly reported wild edible and medicinal plants in the study sites by different informants used the same edible and medicinal plant parts including different medicinal and wild edible used parts. Of these food security edibles, medicinal plant species No. 1, 4, 7–9, and 12–22 were locally extinct plants. Therefore, they need attention for in situ conservation. Here, *M. stenopetala* rarely occurs species in the kola agroclimatic community; it is naturalized in the wild of the study area, and it came from another site. Indigenous people practice growing and diversifying in wild natural habitats to adapt to kola (semidesert) around Gibe River for the source of traditional food security of leaf vegetable as well as local food sovereignty.

### Agroclimatic zones and abundance of WEPs in various habitats

Natural habitats are home to renewable wild edible plants. In the study area, WEPs were gathered from different in situ habitats with various percentages (Table 10). Informants collected more WEPs from wild habitats 59 (92.19%) than non-wild habitats from semi-wilds 5 (7.81%). Large in and around purposively sampled vegetation areas of forest patches and community homegardens even if they were rare due to human activities, mainly deforestation for agricultural expansion and settlements. Few WEP species *C. africana*, *Mimusops kummel*, *S. guineense* var. *guineense*, *S. guineense* subsp. *afromontanum* and *W. ugandensis* are economically very important trees in addition to their uses as wild edible and medicinal plants in the area.

**Table 10:** Ethnobotanical wild edible plants diversity collected in Soro District, southern Ethiopia

No	Vernacular Name	Scientific Name	Family Name	Diversity		Reported wild edible part used	FWEP	Reported medicinal part used	VNo
1	Ashwaala [Wo'o xuuxakkam fiita]	Acanthus sennii Chiov*	Acanthaceae	S	W, GL, D, WD, Co	Sipping/sucking sweet liquid nectar sap	F, Hb, Fin	X	MH-102
2	Hoffi qaccabba	*Achyranthes aspera* L	Amaranthaceae	H	W, HG, D, WD, Co	Leaf with young shoot cooked	Veg, M, Fo	Leaf or whole parts	MH-75
3	Illilli qubba	*Acokanthera schimperi* (A. DC.) Schweinf	Apocynaceae	T	W, FP, WD, Co	Raw ripe dark-red fruit	F, Fw, Pn, Sha	X: Stem; leaf latex poisonous	MH-265
4	Zeqisse [Ajaar jeela laba]	*Ageratum conyzoides *L	Asteraceae	H	W, HG, D, WD, K, Co	Leaves with young shoot cooked	F, M, Fo, Hb	Leaf or whole parts	MH-286
5	Abbara	*Allophylus abyssinicus* (Hochst.) Radlkofer	Sapindaceae	T	W, FP, WD, Ra	Raw ripe orange fruit	F, Fw, Ft, Hb	X	MH-264
6	Haliba	*Amaranthus caudatus* L	Amaranthaceae	H	W, HG, D, WD, Co	Leaf, young shoot, seed cooked	Veg, M, Hb	Leaf, seed	MH-77
7	Gude’e	*Amaranthus dubius* Thell	Amaranthaceae	H	W, HG, D, WD, K. Co	Leaf with young shoot cooked	Veg,M, Fo, Hb	Leaf, young shoot	MH-65
8	Hundufaanna	*Asparagus africanus* Lam	Asparagaceae	H	W, FP, D, K, Ra	Raw ripe orange fruit	F, M	Leaf and root	MH-198
9	Baddanno'o	*Balanites aegyptiaca *(L.) Del	Balanitaceae	T	W, FP, WD, K, Co	Raw ripe yellow fruit	M, Fo, Df, Fw, Ch, Res	Stem charcoal, Resin	MH-16
10	Kashar jeela	*Bidens pachyloma *(Oliv. & Hiern) Cufod	Asteraceae	H	W, HG, WD, K, Co	Young shoot, bud and leaf cooked	Veg, M, Fo	Leaf, young shoot, bud	MH-63
11	Meent alabo	*Bidens pilosa* L.	Asteraceae	H	W, HG, D, WD, Co	Leaf with young shoot cooked	Veg, M, Fo	Ag—aboveground, leaf	MH-82
12	Qaala'i biishsho'o	*Bridelia* sp.	Euphorbiaceae	T	W, FP, WD, K, Co	Light-green leaf, young shoot cooked	M, F, Fo, Fw	Leaf	MH-373
13	Qoqombe'e	*Carissa spinarum* L.	Apocynaceae	S	W, FP, K, Co	Raw ripe dark black fruit	F, M, Fo, Co	Fruit, stem bark, root	MH-328
14	Lob gu’ma	*Commelina benghalensis *L	Commelinaceae	H	W, HG, D, WD, Co	Leaf and young bud	F, M, Fo	Leaf, bud	MH-81
15	Weddeeshsha	*Cordia africana* L.	Boraginaceae	T	W, AL, D, WD, K, Co	Raw ripe yellow fruit	M, F, TP, Ut, Ff, EI	Leaf, bud and stem bark	MH-115
16	Gidiidoola	*Cyphostemma pannosum* Vollesen.*	Vitaceae	H	W, GL, WD, K, Co	Dark raw ripe fruit	F, M	Fresh root, Root bark	MH-330
17	Omoro’o	*Dicliptera laxata *C.B. Clarke	Acanthaceae	H	W, HG, WD, Co	Leaf, young shoot	F, Tsp, M, Hb	Leaf, Young shoot	MH-79
18	Qoxino'o	*Dioscorea schimperiana* Kunth	Dioscoreaceae	Cl	SW, HG, WD, K, Co	Tuber cooked and eaten	F, M	Tuber	MH-315
19	Fuga'i leega [miqqe'e]	*Diospyros mespiliformis* Hochst. ex A. DC	Ebenaceae	T	W, FP, K, Co	Raw ripen fruit	F, Fw, Ch, Con, Ft, Ut	X	MH-352
20	Haqqi wo’l [Doo'm] kooshshima	*Dovyalis abyssinica *(A. Rich.)	Flacourtiaceae	T	W, FP, D, WD, Co	Eaten yellow raw ripen fruit	F, M, Fo, Fw	Raw ripe yellow fruit	MH-170
21	Ooxxi kooshshaama	*Dovyalis caffra* (Hook. f. & Harv.) Hook. f	Flacourtiaceae	S	SW, HG, D, WD, Co	Raw ripe yellow fruit is eaten	F, M, Lf	Leaf or bud	MH-17
22	Qaanqa [ Xuda laba]	*Embelia schimperi* Vatke	Myrsinaceae	S	W, FP, D, WD, K, Co	Eaten raw ripe fruit	F, Fo	X	MH-304
23	Ciishaana	*Erucastrum arabicum* Fisch & Mey	Brassicaceae	H	W, HG, D, WD, Co	Leaf and bud cooked	Veg, M, Fo	Aboveground, leaf	MH-190
24	Meegaara	*Euclea divinorum* Hiern	Ebenaceae	T	W, FP, WD, K, Co	Raw dark black fruit	F, M, Df, Fw, Hb, Fin	Leaf	MH-15
25	Qodde’i oda’a	*Ficus sur* Forssk	Moraceae	T	W, FP, WD, K, Co	Raw ripe orange fruit	F, M, Fo, Am, Gu, Sha, Ut, TP, Fw	Stem bark and latex	MH-289
26	Oda’a	*Ficus sycomorus* L.	Moraceae	T	W, FP, D, WD, K, Co	Raw ripe orange fruit	F, M, Fo, Ut, Gu, Sha, Ut, TP, Am, Fw	Stem bark and latex	MH-98
27	Qaal'i odechcho	*Ficus thonningii* Blume	Moraceae	T	W, Riv, K, Co	Raw ripe fruit	F, TP, Ut, Fw, Am	Stem latex	MH-376
28	Qilxo'o	*Ficus vasta* Forssk	Moraceae	T	W, FP, K, Co	Raw ripe fruit	F, M, Fo, Ut, Gu, Fw, TP, Am	Stem bark and latex	MH-177
29	Hagala	*Flacourtia indica* (Burm.f.) Merr	Flacourtiaceae	T	W, FP, D, WD, Ra	Raw ripe dark brown fruit	F, Fw	X	MH128
30	Shillo'o	*Habenaria decumbens* Thomas & Cribb *	Orchidaceae	H	W, GL, WD, Co	Red crushed tuber is eaten	F, M	Tuber	MH-271
31	Hoomba	*Landolphia buchananii* (Hall.f.) Stapf	Apocynaceae	Li	W, AL, WD, Ra	Raw ripe varied colored fruit	F, M, Cul, La, Con, Ut	Raw ripe fruit	MH-147
32	Qaala'i qashsha	*Gmnosporia senegalensis* (Lam.) Loes. [*Maytenus senegalensis* (Lam.) Exell.].	Celastraceae	T	W, FP, K, Co	Raw ripe fruit	F, Fo, Fw	Leaf/bud	MH-354
33	Dogoo'na	*Mimusops kummel* A. DC. Kumel	Sapotaceae	T	W, FP, D, WD, K, Co	Raw ripe yellow fruit	F, Con, Fw, Ch	X	MH-131
34	Hamashshi waasa	*Momordica foetida* Schumach	Cucurbitaceae	Cl	W, HG, D, WD, Co	Raw ripe fruit	F, M	Raw ripe fruit, leaf, bud	MH-06
35	Haqqi shaana [Moringa]	*Moringa stenopetala* (Bak. f.) Cuf	Moringaceae	T	SW, HG, K, Ra	Leaf with young shoot	Veg, M	Leaf	MH-62
36	Gexeema	*Myrsine africana* L.	Myrsinaceae	S	W, FP, D, WD, Co	Raw ripe fruit	F, Fo, Tb, Cul	X	MH-132
37	Itakkam kuukka	*Oncoba spinosa* Forssk	Flacourtiaceae	T	W, FP, K, Co	Raw ripe dark-brown-colored fruit	F, M, Fo, Fw	Leaf, fruit	MH-351
38	Goro’ama [Cii'i mixmimixo'o]	*Oxalis corniculata* L.	Oxalidaceae	H	W, GL, D, WD,K, Co	Leaf is eaten by cooked	F, M, Hb, Fin	Whole parts, leaf	MH-150
39	Kookile’e	*Passiflora edulis* Sims	Passifloraceae	Cl	W, HG, WD, Ra	Raw ripe dark red fruit	F, Hb, Fin	X	MH-142
40	Sale’e [Dimbaaba]	*Phoenix reclinata* acq	Arecaceae	T	W, Riv, D, WD, Co	Raw ripe orange fruit	F, M, Fo, Con, Orn, Hb, Fin	Leaf, bud/twig	MH-110
41	Onjooro’o	*Physalis peruviana* L.	Solanaceae	H	W, HG, D, WD, Co	Raw ripe orange fruit	F, M, Hb, Fin	Leaf/bud, root	MH-114
42	Macco'i qarra [Qaal’i weddeeshsha]	*Piliostigma thonningii* (Schumach.) Milne*-*Redh	Fabaceae	T	W, FP, WD, K, Co	Leaf cooked roasted; chewed swallow the juice	F, M, Ch, Fw	Leaf, fruit	MH-323
43	Qamo’o	*Rhus vulgaris* Meikle	Anacardiaceae	T	W, FP, D, WD, Co	Light green raw ripe fruit	F, M, Fo, Fw, Ch	Leaf, stem bark	MH-08
44	Dabayyi gora	*Rubus apetalus* Poir	Rosaceae	S	W, RS, D, WD, Co	Black raw ripe fruit	F, M, Fo, Hb, Fin	Leaf	MH-11
45	Daane'i gora	*Rubus steudneri* Schweinf	Rosaceae	S	W, RS, D, WD, Co	Raw ripe fruit	F, M, Fo, Hb, Fin	Bud	MH-41
46	Shiisho’o	*Rumex abyssinicus* Jacq	Polygonaceae	H	W, HG, D, WD, Co	Leaf, young shoot roasted	F, M, Fo	Leaf, root	MH-22
47	Imbocca	*Rumex nervosus* Vahl	Polygonaceae	S	W, Lf and Df, D, WD, Ra	Roasted leaf, young shoot	F, M, Fo	Leaf, root	MH-307
48	Gaarawwa	*Rytigynia neglecta* (Hiern.) Robyns	Rubiaceae	S	W, Lf, D, WD, Co	Raw ripe deep black fruit, sweet tested	F, M, Fo	Leaf, stem bark	MH-48
49	Faraxxi qasa	*Sideroxylon oxyacanthum* Baill	Sapotaceae	T	W, FP, D, WD, Ra	Raw ripe fruit	F, M, Fo, Fw, Df	Leaf, bud, stem bark	MH-28
50	Heemachchi migillo’o	*Solanum nigrum* L.	Solanaceae	H	W, HG, D, WD, Co	Aboveground, leaf cooked /raw ripe	F, M, Fo, Hb	Leaf/bud	MH-13
51	Bulo’o	*Solanum* sp.	Solanaceae	S	W, HG, WD, Ra	Leaf, young shoot cooked	Veg, M, Hb	Leaf	MH-182
52	Ajaar migillo'o	*Solanum villosum* Mill	Solanaceae	H	W, AL, D, WD, K, Co	Leaf, bud, ripe fruit	Veg, M, Fo, Hb	Leaf, bud	MH-336
53	Duubaana	*Syzygium guineense* (Wild.) DC. Var.* guineense*	Myrtaceae	T	W, Ria, WD, K, Co	Raw ripe different colored fresh fruit	F, M, Con, Ut, Df, Sha, Hb, Fin	Leaf, bud, fruit	MH-317
54	Gooto'i duubaana	*Syzygium guineense (*Willd.) DC. subsp. *afromontanum* F. White	Myrtaceae	T	W, D, FP, WD, Co	Raw ripe different colored fruit	F, M, Con, Ut, Df, Sha, Hb, Fin	Leaf, bud, fruit	MH-171
55	Guff haata	*Thunbergia ruspolii *Lindau*	Acanthaceae	H	W, GL, WD, Co	Leaf with young shoot cooked	M, F, Fo	Whole part, leaf	MH-308
56	Ishinna	*Thymus schimperi* Ron.*	Lamiaceae	H	SW, HG, D, WD, Co	Leaf, bud as spices	F, Sp, M		Leaf, bud
57	Seego, o	*Toddalia asiatica *(L.) Lam	Rutaceae	Cl	W, RS, D, WD, Co	Raw ripe orange fruit	F, M, Fo	Fruit, leaf, bud	MH-09
58	Qabarbuyya	*Trichocladus ellipticus* Eckl. & Zeyh	Hamamelidaceae	S	W, Riv, WD, K, Co	Matured raw leaf	F, M, Fo, Fw, Hb, Fin	Leaf, bud	MH-78
59	Cimcima	*Urtica simensis* Steudel.*	Urticaceae	H	W, Riv, D, WD	Leaf with bud cooked	Veg, M	Leaf/bud	MH-194
60	Lob doobba	*Urtica urens* L.	Urticaceae	H	W, HG, D, WD, Co	Leaf with bud cooked	Veg, M	Leaf, bud, root	MH-58
61	Loqe'e	*Vangueria volkensii *K. Schum	Rubiaceae	S	W, FP, WD, K, Co	Raw ripe dark fruit	F, Fw	X	MH-311
62	Leega	*Warburgia ugandensis* Sprague	Canellaceae	T	W, Riv, WD, K, Ra	Raw ripe light-green spherical fruit	F, Con, Ft, Cul, Ut, Po	X	MH-269
63	Qaal’i kooshimma	*Ximenia americana* L.	Olacaceae	S	W, FP, K, Co	Raw ripe yellow fruit is eaten	F, Fo, M, Fw, Con	Fruit, Leaf, Stem bark, Root bark	MH-273
64	Gaaq xuranqa	*Ziziphus spina-christi* (L.) Desf	Rhamnaceae	T	W, FP, WE, K, Co	Raw ripe fruit	M, F, Fo Fw	Leaf, fruit	MH-359

Vernacular name, Scientific name, Family name, Habit = Hab, Diversity, Function of wild edible plant = FWEP, reported medicinal and wild edible part used; Voucher No/VNo: MH-XY/XYZ [2–3 digital number; MH, Mulatu Hankiso; XY, two digital number; XYZ, three digital no.]

*W* Wild, *SW* Semi-wild, agroclimatic zone: *D* Dega, *WD* woinadega, *K* kola, *M* Medicine, *F* Food, *Fo* Fodder/forage, *Veg* vegetable, *MSp* both medicinal and spice, *Tsp* Tea spices, *MF* Both medicinal and food, *EI* Economic income, *Cul* Cultural, *Fw* Fire wood, *Po* Pole, *Tb* Toothbrush, *TP* Timber production, *Pn* poison, *Hb* honeybee, *Fl* flower–inflorescence-nectar = Fin or flower, *St-ch* Stem charcoal, *Con* Constructions, *Ut* utensils, *Ft* Farming tool, *Orn* Ornamental, *Sha* shade, *Ff* Fire formation, *Lf/&Df* Live fence/&dry fence, *Am* Attachment); functional part: *Wh* (aboveground = Ag or belowground = Bg) whole part, *Ysh* Young shoot, *L/Bd* Leaf/bud, *R* Root, *RL* Root&leaf, *Tu* tuber, *St* Stem, *Fr* fruit, *Se* Seed, *Sb* stem bark, *Rb* Root bark, *La* Latex, *Sa* Sap, *Res* Resin), *GL/BL* Locality: grass/bush land, *Riv* Riverine, *RS* Roadsides, *FP* Forest patch, dominance: common = Co; *Ra* Rare, endemic plant species(*). *Hab* Habit, Diversity, *FWEP* Function of wild edible plant, *X* Not reported for medicinal use

### Threats and conservation strategies of wild edible plants

In the study area, human activities (anthropogenic factors) are the main threats to vegetation which causes the decline of multi-purpose indigenous wild food plant species. Deforestation is one of the leading impact factors due to the new settlement and agricultural expansion. Cutting/illegally hunting trees and shrubs from remnant forest patches, grass, riverine and bush lands unwisely for fire and selling local charcoal, timber/furniture production, dry fence, house construction, and *Eucalyptus* trees substitution are also threats that decrease potential vegetation species that provide wild edible plants for food security. For example, *C. spinarum*, *C. africana*, *F. sur*, *F. sycomorus*, *D. schimperiana*, *L. buchananii*, *O. spinosa*, *P. thonningii*, *S. guineense* var. *guineense*, *S. guineense* subsp. *afromontanum, W. ugandensis* and *X. americana* need conservation priority in the community. Overgrazing in the protected vegetation areas, lack of attitudes toward bare land replantation to form afforestation, less knowledge share for a young new generation, and changing climatic condition in the environment also contributes to the threats.

Focus group discussions in the district in the 13 sites of the study kebeles, above various threats, were identified and discussed and followed by suggestions for solutions to conserve and manage those indigenous potential wild edibles and/or medicinal plants in the community, which help to conserve more other potential plants including wildlife in their natural habitats. Mainly in situ conservation of plants in their natural habitats as well as ex situ conservation and awareness education for communities. Domestication of indigenous potential wild edible and medicinal plants by local people around home guards, agricultural land, roadsides, shade, nursery expansion, reducing exotic plantation (e.g., *Eucalyptus* trees), reforestation, and afforestation. Hence these strengthening conservation strategies of vegetation (remaining forest patches) in the study area. These are with the help of nearby governmental institutes with community linkage.

## Discussion

Most of the gathered and identified WEPs are used by the people of the study area for various purposes in addition to being used as wild food sources, contributing to food security in a similar way to other parts of Ethiopia and the rest of Africa. A relatively diverse number of WEPs with dominant families were documented from various agroecological zones and habitats. The number of species recorded is higher than the findings reported by some of the studies made in Ethiopia [4, 12, 15, 25, 40, 41] and Uganda [42]. Various WEPs, plants with different habits and edible parts were documented and compared with data from other parts of Ethiopia. For example, indigenous fruit trees of *P. reclinata* and *Rhus vulgaris* were reported as potential economic plants in Mukoro District, Uganda [43]. These are also potential wild-growing trees with parts consumed by the people of Soro District. This current study contributes a taxonomically varied 64 species distributed in 52 genera and 39 families; which is by far closely comparable with the findings of Amente [12] which reported 60 species in 49 genera and 35 families. Moreover, this study has added 16 new species to the existing records/database of Ethiopian WEPs. Flacourtiaceae, Solanaceae, and Moraceae were the dominant families that contributed the highest number of WEPs in this study. The Moraceae contributed to four important wild edible fruit trees in addition to their promising nutritional values as reported by Tebkew et al. [4] and Dejene et al. [14]. The Myrtaceae and Rosaceae contributed two nutritionally useful species in agreement with previous works [12]. Similarly, the two WEPs of the Myrtaceae reaffirm the findings of Demise [41] on the ethnobotanical study of WEPs in the Adola District, southeastern Ethiopia. Our results are generally comparable with findings from some other districts [4, 12, 19, 24–26, 44, 45]. However, the study by Addis et al. [18, 23], Balemie and Kebebew [9] in Ethiopia and another study from Western Nepal [46] reported higher numbers of WEPs than the current study. The variation could be related to the agroclimatic differences, the size of the study areas, the cultural settings and the research intensity. The overall assessment showed that Soro District maintains a rich assemblage of wild edible and nutraceutical plant diversity and associated ethnobotanical knowledge.

Some multifarious WEPs were commonly reported from different study areas in Ethiopia including 25 species from Chelia District, West-Central Ethiopia [26]; 23 species in Berehet District, North Shewa Zone of Amhara Region [20];16 species in Central Ethiopia [47]; 16 species in Konso Ethnic community, southern Ethiopia [23]; 15 species in Burji District, Segan Area Zone [25]; 14 species in Chilga District, northwestern Ethiopia [48];14 species in Kamashi Wereda Benishangul Gumuz Region [12]; 12 species in Quara District, northwest Ethiopia [4]; 10 species in Western Nepal [46]; 9 species in Benna Tsemay District [49] and 8 species in Derashe and Kucha Districts of South Ethiopia [9]; 7 species in Uganda [42]. Thus, the abundance of WEPs observed in the present study area is shared with numerous distribution ranges in different agroecological zones of Ethiopia [9, 48, 50]. The provenance of these WEPs in various growing habitats increases the population of species and the culture of traditional knowledge with plant diversity [48]. Of the growth habits, trees were dominantly used life forms in the study area that strongly support the agreement of [4, 10, 24, 40]. This habit proportion is in contrast with Lulekal et al. [17] in southern Ethiopia, and Godfrey et al. [51] in the Bunyoro Kitara kingdom of Uganda reported that shrubs and herbs were the dominant habits contrasting with the dominant growth form (trees) found in the current study of Soro District [12]. Similarly, trees and herbs were reported to have the highest parts consumed, followed by shrubs in agreement with Kidane et al. [24] which pointed out trees were consumed more by the Maale and Ari ethnic groups in southern Ethiopia. Different habits of WEPs in three agroclimatic zones and vegetation features of the study area contrast with the discussion of Ashagre et al. [25].

Plants with edible fruits contributed the largest proportion of 34 (53.13%) species having parts eaten and mostly used as a ripe raw form, which agrees with the findings of other works [14, 20, 23, 40, 41, 47], also leaves follow as the second largest group of edible part accounting to 19 (29.69%) species in agreement with some studies [12, 23]. Wide consumption of fruits has been reported in many studies [4, 14, 17, 18, 22, 23, 40, 41, 48, 50] investigated in different parts of Ethiopia. Leaves were also among the widely reported edible parts [9, 11, 12, 17, 25, 41]. Most WEPs of Soro District are consumed raw when ripe without processing and cooked/roasted similar to the reports of other researchers [18, 23, 24]. During the study, about 44 (68.75%) species were observed while being eaten raw upon simple processing by cleaning dirty materials, washing the edible parts, and removing thick or thin inedible exocarp in the case of fruits, and some endocarp parts (hard stone seed/s) but 19 edibles (29.69%) were eaten by chopping with a knife after roasting or cooking using local clay pots, metallic cookers. Stems and leaf parts were the most used plant parts in the northern West Bank of Palestine [52].

Parts of most WEP (fruits/leaves) are eaten raw and support community members that need snacks or emergency foods [11, 15, 41]. Among species that have edible fruits, the stem latex is used by injuring or cutting the bark of the stem part for releasing out the latex, which sipped/sucked by children looking after domestic animals, also used as chewing gum by painting or smearing the milky latex on the hand and let it dry (e.g., stem latex of *L. buchananii*, *F. sycomorus,* and *F. sur*). *Carissa spinarum*, *F. sur* and *X. americana* were most frequently preferred edible wild edible fruits during shortages of regular food. Similarly, Kidane et al. [24] reported in the Debub Omo Zone of southern Ethiopia that these species were among the most widely harvested wild edible fruits during food shortages/famines due to drought. Among 52 wild and semi-wild dietary ethnobotanical fruits used by the Maale and Ari ethnic communities in southern Ethiopia [24], 11 fruits are also found in Soro District. Of these seasonally available fruits, *Z. spina-christi* and *D. mespiliformis* contribute essential nutrients for Maale and Ari communities [24] and also contributed higher amounts of nutrient contents that provide the guaranteed availability than cultivators as described by Mengistu and Hager [50]. In the community among the discussed, ordered and ranked WEPs and preferential and selective plant species reported by most informants (Tables 3, 4, 5, 6 and 7) as significant species, and they are potential plants for food security as well as food sovereignty in the study area and include *F. sycomorus, L. buchananii, F. sur, W. ugandensis, S. guineense* var. *guineense*, *S. guineense* subsp. *afromontanum*, *O. spinosa*, *X. americana, D. schimperiana* and *Habenaria decumbens* reported from three different agroecologies. Some species among the leafy vegetables with young shoots with buds and fruits are preferred by some. The species *S. nigrum, A. dubius,* and *B. pachyloma* were among those preferred by most key informants as leafy vegetables and the most potential species to secure food during food shortage or unavailability.

These selectively eaten and other plant species were used for health care as sources of traditional medicine showing that the borderline between wild edible plants and traditional herbal medicine is not that sharp. Similar findings were reported by other researchers [20, 53]. Moreover, the above-mentioned species are fruits, leaves and young shoots, and tuber edibles that are very essential plant species in the community to give different foodstuffs, mineral contents, antioxidants, and vitamins.

Among the total collected wild edible plants, some are used for both food and medicinal value with the same part being used for food and medicine; local informants also reported others used with different edible and medicinal parts to treat various human and livestock ailments (Table 9) in the ailments category of dermatological, gastrointestinal, haem parasitic, circulatory, endocrine system, orbital, cardiovascular, glandular, dental, digestion, external and internal cancer, respiratory, intestinal parasitic, protozoan, systemic and urinary tract system (UTS). The medicinal worth of nutraceutical plants lies in the fact that in most cases toxicity issues have already been pretested culturally.

Particular wild edible fruits, *B. aegyptiaca, C. spinarum, X. americana and Z. spina-christi* were highly cited in kola agroclimatic kebeles where they occurred and consumed widely; mostly as critical supplementary fruit food species. Excluding *C. spinarum,* this finding agrees with Tebkew et al. [4], but *Z. spina-christi* showed low informant citations in Soro District (Table 10).

The highest frequency occurred for *F. sycomorus* in the case of informant consensus and SPR and FL in the current study and highly cited [4]; similarly, *S. guineense* var. *guineense* was recorded relatively higher in the SPR and DMR, which is in line with other studies [48, 59], and contrastingly lower in another study [4]. However, insufficient informant citations are noted in other study areas [50, 59]. The species citation was varied in the study area because it depends on informants’ knowledge between communities of various study sites in agreement with the report by Tebkew et al. [4]. The above species are locally prioritized traditional wild food species as shown by the current study, like in other parts of Ethiopia and some African countries such as Kenya, Sudan, and Tanzania [4].

As the index of FL, *F. sycomorus*, *A. dubius*, *C. spinarum*, and *S. guineense* var. *guineense,* and *S. guineense* subsp. *afromontanum* were potential wild edibles in the study area; similarly, the latter two species shared their use values [25] in Burji District, in a few rural sites as the source of income generation [59]. Likewise, in the study area, indigenous fruits of *S. guineense* subsp. *afromontanum* and *S. guineense* var. *guineense* before decades were sold as dietary food and generated income in local markets. Nowadays, either these species or other WEP species are not targets of selling for income sources. They are commonly sold in other parts of Ethiopia as wild food while our findings differed from another study [60].

Socio-demographic features and cultural attributes of the current study showed variations with relatively different indigenous knowledge and traditional practices in the use of wild edible plant. Adults cited a higher number of WEPs than youngsters, which may be due to differences in experience and knowledge gap on WEPs in the current study, which came up with insignificant difference (*P* > 0.05); similarly, adults cited more WEPs than youngsters in another study [4]; still in another study, youngsters reported more than adults [61]; this may be due to less attention from adults as a result of being dependent on the modern food system. An average number of male interviewees were more knowledgeable, quoted the highest proportion of WEPs than females, and this is highly significant (*P* < 0.05) in contrast with one study [26] that showed women reported more plant species than men. Key informants reported significantly higher mean average number of wild edible species than general informants (*P* < 0.05) which agrees with one study [26].

On the contrary, males had less practical knowledge than females on wild food preparation; frequently literates reported more WEPs than illiterates, general informants reported more WEPs than the key informants but may be less retained indigenous knowledge than key informants. Informants cited more WEPs in woinadega than kola and dega, and in contrast one other study [48]. Elderly informants were better at generating inherited information and longtime retained use experiences than youngsters in the current study in a similar way to the findings of another study [26].

Rural dwellers reported more wild edible plants than urban dwellers in the study area. The findings are in contrast to other results [50, 59], and are similarly reported in the world [60, 62]. However, more WEPs were frequently reported from far away (> 5 km, about 75.03%); less percentage from proximate to the main town (< 5 km, approximately 24.97%). This indicated that more varieties of WEPs were collected/available in a rural site near and in forest patches of the study area than the town proximities. Seven of the WEPs (*Acanthus sennii*, *B. pachyloma*, *C. spinarum*, *H. decumbens*, *Thunbergia ruspolii*, *T. schimperi*, and *Urtica simensis)* are unique plants endemic to Ethiopia also known to be used in the Ethiopian traditional herbal healing system in the study area. Six of the endemic WEPs were similarly reported earlier [63]; and two of them (*U. simensis* and *T. schimperi)* were reported by another study [49].

In the study, *C. africana*, *S. guineense* var. *guineense*, *S. guineense* subsp. *afromontanum,* and *W. ugandensis* are economically significant income source tree species in the community, in addition to their nutraceutical values (Table 5). Among the WEPs of Soro District, commercially important and edible fruit-yielding tree species are also reported from other parts of Ethiopia [64]. As such species are needed for timber, they are highly vulnerable and they are prioritized for strong conservation attention.

Many WEPs and other biodiversity components in the study area are affected by various threatening factors as in other parts of Ethiopia [4, 9, 14, 17, 48]. Findings of the impacting factors are in agreement with other investigations [12] in that agricultural activities and climate fluctuations/variability that result in drought and lead to famine are the most threatening factors. Deforestation, construction material extraction, overgrazing by livestock, and collection of fuel wood usually by selective cutting were the leading causes of the loss in the study area which was similarly reported in another study [59]. Another researcher [8] reported drought that led to the famine accounting for the major factor that resulted as a consequence of the collection of fuel wood for firewood collection and charcoal making, timber for construction, and dry fence. These uses collectively decreased the availability, affordability, diversity and the number of taxa in the study area. Affordability, availability and accessibility, and utilization of WEP species have been faced with many challenging factors in agreement with findings by other researchers [9]. Utilization of WEPs is faced with challenges related to ripening or maturity time, collection time and keeping the quality (shelf life) of the production without spoilage due to decaying. These are some of the reasons for local people in giving less attention to wild edibles as supplementary food sources. The paucity in cultural awareness and perception about yields and the benefits of the nutritional values of wild edible plants adds to the challenges.

The mode of consumption of wild edible plants is affected by various factors such as climatic (environmental) factors under which they grow, time of ripening and difficulties for harvesting, and rapid deterioration within a short time thereby decreasing their quality and food values [4, 40]. These factors are thought to be reasons behind people’s reduced attention to wild edibles, thus the tendency to rely more on stable food sources [9, 24]. Fast destruction of WEPs may be caused as a result of inappropriate collecting (harvesting) practices for various functions.

Various WEPs and nutraceutical as well as antioxidant plants, for instance, *C. spinarum*, *X. americana* and *C. africana* are exposed to human pressure in the study area as also from studies undertaken by [20]. The limited conservation and management considerations for these and other multi-functional WEPs are leading to erosion/loss of plant resources. This increases the disappearance or local extinction of useful plant species from different habitats; the consequence tends to lead to the loss of indigenous knowledge, making conservation and management of the remaining vegetation of Soro District very crucial. Thus, planting multiuse WEPs round the home yards, grasslands, fence lines and farming lands is helpful for easy access to nutritional, economical, medicinal and environmental benefits to the community. In addition, it saves and secures indigenous and endemic plants from extinction and wild food sources to combat future occurrences of drought and famine. Moreover, it would strengthen the ecological and ethnobotanical sustainability of the study area from the loss of natural resources and would contribute to the augmentation of the livelihoods of the local people.

## Conclusion

This study documented 64 wild edible plant species as the source of wild food plants. Through the investigation into sampled kebeles (*n* = 13), only one poisonous species (*A. schimperi*) was recorded as perceived by the local people, but not in its edible part. The people reported their use of the stem of this species for making arrow poison to track down wild animals, further indicating that the leaves are as poisonous being toxic or lethal when eaten. Different habitats provide wild edible plants to the community, and many species are consumed as supplementary food at any normal time and during food insecurity, also for medicinal values and multi-functional uses. Wild trees and herbs with edible parts came up with more species than shrubs, climbers and liana in that order. Fruits were highly accepted and preferentially consumed by the community as raw ripe forms. Indigenous people of the district use few WEPs from their private holdings and more of them from potential vegetation sites that retain forest patches. Other parts are picked and consumed as leafy vegetables, roots/tuber, chewing gum, sucking flower sap and tea spices, also essentials for herbal medicine and other services.

The preferences of wild edible plants using informant consensus, ICF, preference ranking, direct matrix ranking, paired comparison, and index of fidelity level gave clues to the need for conservation priority attention through in situ as well as ex situ strategies. In addition, the domestication of multi-functional WEPs used in connection to many anthropological activities in their natural habitats and within home gardens, agricultural lands, shades, and cultural areas is important for the community. Such actions and strategies are essential for the conservation of the wealth of various plant species which increase the affordability and accessibility of wild edibles and nutraceutical plants. Furthermore, it is useful to conserve wildlife and keep the ecological balance in the environment.

The findings of this study indicated that conservation training actions for multipurpose indigenous biodiversity by giving priority and increased attention to the declining species and those on the verge of extinction. Educational training workshops targeting communities need to be considered among the solutions with the collaboration of nearby institutions and agricultural offices. It helps to take conservation measures against anthropological activities which encourage saving indigenous plants with the association of wildlife in their habitats as well as giving environmental advantages.

## Data Availability

All the data used to support this study are included in the paper and available in the supplementary material.
